# Changes in Air CO_2_ Concentration Differentially Alter Transcript Levels of *NtAQP1* and *NtPIP2;*1 Aquaporin Genes in Tobacco Leaves

**DOI:** 10.3390/ijms17040567

**Published:** 2016-04-14

**Authors:** Francesca Secchi, Andrea Schubert, Claudio Lovisolo

**Affiliations:** Dipartimento di Scienze Agrarie, Forestali e Alimentari (DISAFA), ULF Colture arboree e Fisiologia Vegetale, Largo Paolo Braccini 2, 10095 Grugliasco (TO), Italy; andrea.schubert@unito.it (A.S.); claudio.lovisolo@unito.it (C.L.)

**Keywords:** carbon dioxide (CO_2_), *NtAQP1*, *NtPIP2;1*, aquaporin, photosynthesis, stomatal conductance, *Nicotiana tabacum*, gene expression

## Abstract

The aquaporin specific control on water *versus* carbon pathways in leaves is pivotal in controlling gas exchange and leaf hydraulics. We investigated whether *Nicotiana tabacum* aquaporin *1* (*NtAQP1*) and *Nicotiana tabacum* plasma membrane intrinsic protein 2;1 (*NtPIP2;1*) gene expression varies in tobacco leaves subjected to treatments with different CO_2_ concentrations (ranging from 0 to 800 ppm), inducing changes in photosynthesis, stomatal regulation and water evaporation from the leaf. Changes in air CO_2_ concentration ([CO_2_]) affected net photosynthesis (Pn) and leaf substomatal [CO_2_] (Ci). Pn was slightly negative at 0 ppm air CO_2_; it was one-third that of ambient controls at 200 ppm, and not different from controls at 800 ppm. Leaves fed with 800 ppm [CO_2_] showed one-third reduced stomatal conductance (g_s_) and transpiration (E), and their g_s_ was in turn slightly lower than in 200 ppm– and in 0 ppm–treated leaves. The 800 ppm air [CO_2_] strongly impaired both *NtAQP1* and *NtPIP2;1* gene expression, whereas 0 ppm air [CO_2_], a concentration below any *in vivo* possible conditions and specifically chosen to maximize the gene expression alteration, increased only the *NtAQP1* transcript level. We propose that *NtAQP1* expression, an aquaporin devoted to CO_2_ transport, positively responds to CO_2_ scarcity in the air in the whole range 0–800 ppm. On the contrary, expression of *NtPIP2;1*, an aquaporin not devoted to CO_2_ transport, is related to water balance in the leaf, and changes in parallel with g_s_. These observations fit in a model where upregulation of leaf aquaporins is activated at low Ci, while downregulation occurs when high Ci saturates photosynthesis and causes stomatal closure.

## 1. Introduction

Aquaporins are a family of small pore-forming integral membrane proteins that play an important role in plant water relations by facilitating water transport along a water potential gradient [1,2]. The physiological relevance of these proteins for transmembrane water flow has been hinted in experiments where their function was blocked by the inhibitor mercury chloride [3] and it has been more convincingly demonstrated using plants where the expression of selected aquaporins was inhibited or enhanced following genetic transformation [4,5,6,7].

A new perspective in the study of these proteins has been opened by observations that some members of the family do not facilitate water transport [8], or are able to transport other neutral molecules beside water, the most physiologically important among them being CO_2_ [9,10]. The first indirect evidence that CO_2_ may permeate aquaporins in plants was provided by Terashima and Ono [11], who showed that treatment with mercury chloride on *Vicia faba* and *Phaseolus vulgaris* leaves limits mesophyll CO_2_ conductance. Evidence that the tobacco aquaporin *Nicotiana tabacum* Aquaporin 1 (NtAQP1) facilitates transmembrane CO_2_ transport was provided by Uehlein *et al.* [12] using tobacco plants with altered aquaporin expression and injection in *Xenopus laevis* oocytes. In addition, the same authors confirmed the role of *NtAQP1* as chloroplast gas pore: a low CO_2_ permeability of the inner chloroplast membranes was measured in plants where the *NtAQP1* expression was repressed [13].

Analyses of the role of aquaporins in CO_2_ transport in leaves were performed by Hanba *et al.* [14] in rice, by Flexas *et al.* [15] in tobacco and by Hechwolf *et al.* [16] in *Arabidopsis*. Furthermore, the use of reverse genetic approaches clearly demonstrated that inhibition of plasma membrane intrinsic protein 1 (*PIP1*) gene expression determined lower mesophyll conductance to CO_2_ in both *Arabidopsis* [17] and poplar [6] transgenic plants. All these studies strengthen the hypothesis that aquaporins facilitate CO_2_ transport through plant membranes, and suggest that expression and activation of these “CO_2_ porins” may be a significant component of the leaf mesophyll conductance to CO_2_ [18].

In tobacco, two aquaporin genes belonging to the PIP1 and plasma membrane intrinsic protein 2 (PIP2) subfamilies have been isolated and functionally characterized. The *NtAQP1*, a member of the PIP1 subfamily, is expressed in the spongy parenchyma of tobacco leaves, in particular in cells surrounding stomata [19,20]. The PIP2 gene *NtPIP2;1* is expressed in the floral tissue [21], and it plays an important role in water transport in roots [22,23], but its expression in leaves has not been tested up until now. The membrane water permeabilities of *Xenopus* oocytes expressing NtAQP1 and NtPIP2;1 have not been compared directly, but NtPIP2;1 enhances membrane permeability significantly more than NtPIP1;1, another PIP1 aquaporin of tobacco which shows 99% sequence homology with NtAQP1 [21], and NtAQP1 enhances permeability less than other PIP2 aquaporins [20]. In addition, heterologous expression in yeast cells revealed that NtAQP1 did not increase water transport activity, whereas NtPIP2;1 behaved as an efficient water channel [24]. In contrast, facilitation of CO_2_ transport, measured either through enhancement of permeability of *Xenopus* oocyte membranes or by heterologous expression in yeast cells, has been demonstrated for NtAQP1, whereas, on the contrary, NtPIP2;1 lacks a CO_2_-related function [12,24].

While CO_2_ concentration in the atmosphere surrounding leaves is fairly constant in the short term, the concentration within the leaf changes widely due to the combined effects of CO_2_ consumption by carboxylation and CO_2_ production by respiration and photorespiration. It is thus not surprising that, besides its metabolic role as a substrate for RUBISCO and other carboxylases, CO_2_ has a signalling role in plants, inducing physiological effects and, more notably, stomatal closure [25]. Furthermore, the progressive rise in atmospheric CO_2_ concentration is prompting interest in the study of the effects of changes in air [CO_2_] on plants, with the aim of modelling growth and production of plants in future climatic scenarios [26].

Mesophyll conductance to CO_2_ (g_m_) is routinely measured based on a combination of gas exchange and chlorophyll fluorescence techniques. Several environmental conditions such as light intensity and environmental stresses can affect g_m_ [27]. Among other factors, g_m_ is affected by ambient and leaf intercellular CO_2_ concentrations, showing both short-term and acclimation responses [28]. Taking into consideration that aquaporins facilitate the transport of CO_2_ beside water in the mesophyll cells [17,29,30], the question arises whether the regulation of g_m_ by CO_2_ concentration may be mediated by changes in aquaporin expression or activity.

The goal of this work was to investigate whether different CO_2_ concentrations affect the gene expression of two tobacco aquaporins. We choose to study *NtAQP1* because of its proven capacity to transport CO_2_ and because of its relatively low ability to transport water when expressed in *Xenopus* oocytes or yeast cells, and *NtPIP2;1* which, in contrast, is characterized by large water transport rates and no CO_2_ facilitation in the same systems. In our *in planta* system, we show that gene expression for *NtAQP1* positively responds to CO_2_ scarcity in the air, and on the contrary, gene expression for *NtPIP2;1* is possibly related to water balance in the leaf, changing in parallel with stomatal conductance.

## 2. Results

### 2.1. Leaf-to-Air Gas Exchange Responses to CO_2_ Enrichment and Impairment

**Figure 1:**
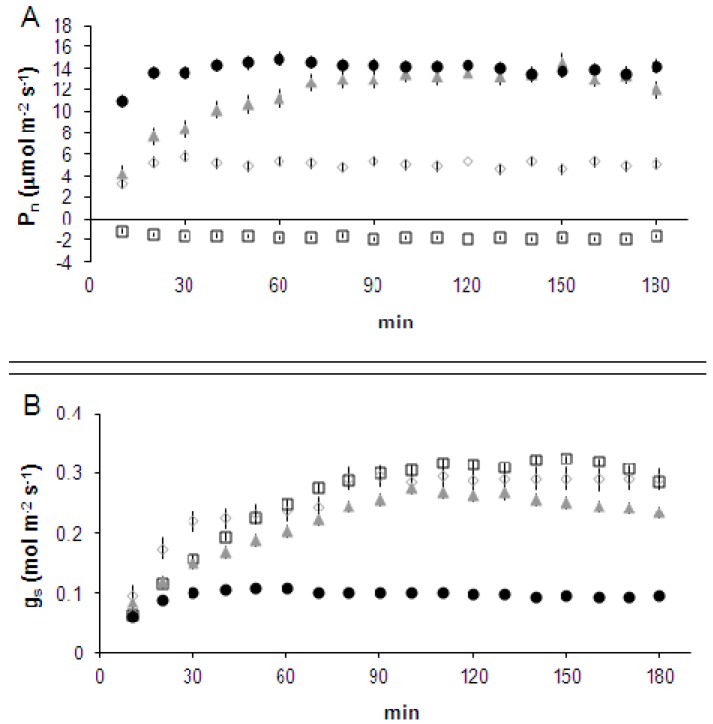

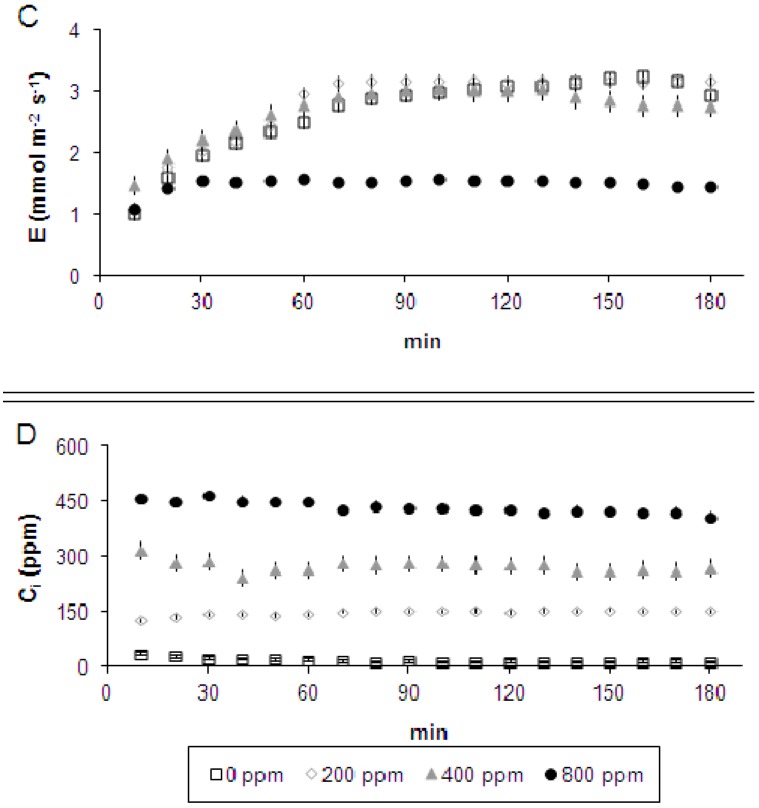
Time course (10 min step) of (**A**) leaf net photosynthesis (P_n_); (**B**) leaf stomatal conductance (g_s_); (**C**) transpiration rate (E); and (**D**) leaf substomatal CO_2_ concentration (C_i_), measured in *Nicotiana tabacum* leaves treated with air containing different CO_2_ concentrations. Zero ppm CO_2_: black empty squares; 200 ppm CO_2_: grey empty diamonds; 400 ppm CO_2_: grey filled triangles; 800 ppm CO_2_: black filled circles. Data are means of five points recorded every two minutes. Data are the averages of three independent biological samples (*error bars* denote SE) for each treatment and time (*n* = 3).

Leaf portions enclosed in a sealed leaf chamber were exposed for up to three hours to air CO_2_ concentrations of, respectively, 0, 200, 400 and 800 ppm. Measurements taken in each of the three treatment periods did not significantly differ between each other at any time of measurement. After a short initial oscillation phase, the concentration of CO_2_ within the leaf chamber remained stable throughout the treatment period at values corresponding to those imposed, within ±3%. Leaf gas exchanges showed a period of adaptation for all treatments, as photosynthetic photon flux density (PPFD) in the greenhouse environment was about one-third that within the leaf chamber, and for this reason stomatal conductance (g_s_) and net photosynthesis (P_n_) were low. At ambient [CO_2_] (400 ppm), the substomatal concentration (C_i_) decreased within the first 30 min to a stable value of 275 ± 2.0 ppm. In this treatment g_s_ increased during the first 90–100 min and slightly decreased during the following 80–90 min, inducing a similar pattern for leaf transpiration (E). P_n_ showed a trend similar to g_s_ but the initial adaptation period ended after about 70 min, *i.e.*, about 20 min before the end of the g_s_ increase, in agreement with an expected metabolic feedback control of stomatal conductance. When leaves were fed at 800 ppm CO_2_, C_i_ was significantly higher than in control leaves (436 ± 2.0 ppm) and, as a consequence, maximum P_n_ was already reached in about 10 min. However, the increase in C_i_ also affected g_s_, which, after a brief increase, remained at about one-third that of ambient controls. As a consequence, even by doubling CO_2_ availability, we observed carbon assimilation values quite similar to those in ambient conditions. Following feeding 200 ppm CO_2_, C_i_ remained stable throughout the experiment at 150 ± 1.0 ppm, and P_n_ was about one-third of the maximum values recorded at both 400 and 800 ppm CO_2_. Although this is expected to release the C_i_ limitation of stomatal opening, g_s_ was not significantly higher than in ambient controls, suggesting that maximum g_s_ values recorded at both ambient and 800 ppm CO_2_ could not be exceeded, as limited by the PPFD. Water loss from the leaf, as estimated by E measurements, was similar in this treatment as compared to 400 ppm CO_2_. Feeding leaves with air containing zero CO_2_ induced a C_i_ very close to zero (19.1 ± 0.7 ppm). Also, in this case, g_s_ did not increase more than observed at 200 ppm CO_2_ while P_n_ was, as expected, lower than zero. Transpiration followed the pattern dictated by g_s_, as in the 400 ppm CO_2_ treatment (Figure 1).

### 2.2. Expression Analysis of NtAQP1 and NtPIP2;1

**Figure 2:**
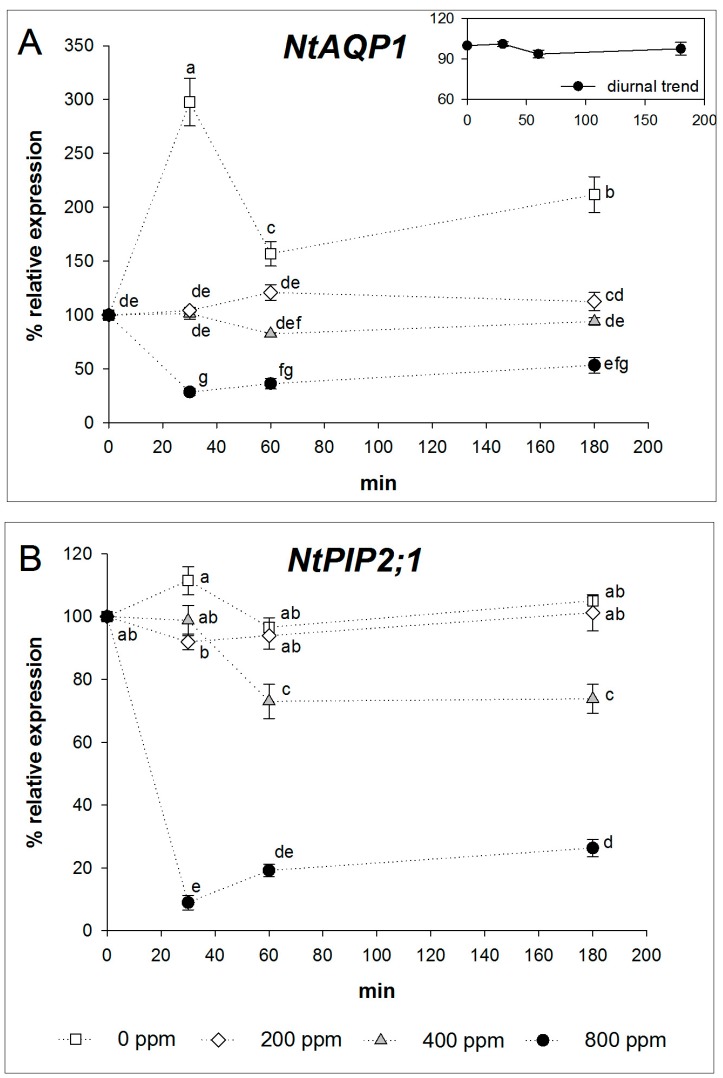
Time course of (**A**) the *Nicotiana tabacum* aquaporin 1 (*NtAQP1*) transcript level and (**B**) the *Nicotiana tabacum* plasma membrane intrinsic protein 2;1 (*NtPIP2;1*) transcript level in tobacco leaves treated with air containing different CO_2_ concentrations. Samples were taken at 0, 30, 60 and 180 min after starting treatment. Values represent expression relative to that observed in control plants (400 ppm CO_2_) at time 0. In the **A** inset, the expression level of *NtAQP1* at ambient [CO_2_], during a time span of 4.5 h (from 10 a.m. to 2:30 p.m.) is displayed. The expression levels of the target genes were normalized using Elongation factor 1 α as internal control. Zero ppm CO_2_: black empty squares; 200 ppm CO_2_: grey empty diamonds; 400 ppm CO_2_: grey filled triangles; 800 ppm CO_2_: black filled circles; samples not subjected to imposed CO_2_: black filled triangles. The results are the averages of three independent biological samples (*error bars* denote SE) for each treatment and time (*n* = 3). Different letters denote statistically significant differences by Tukey’s test.

Since it has been reported that the expression of aquaporins is under circadian regulation [31,32], we preliminarily monitored the expression level of *NtAQP1* at ambient [CO_2_], during a time span of 4.5 h (from 10 a.m. to 2:30 p.m.). No transcript level difference among time points was observed (Figure 2A, inset).

*NtAQP1* gene expression remained fairly constant after 30, 60 and 180 min of 400 ppm CO_2_ treatment. Treatment with 200 ppm CO_2_ induced a slight increase above control in transcript levels after 60 and 180 min from the start of experiment.

A marked and significant increase in *NtAQP1* expression was observed in leaves treated with 0 ppm CO_2_: transcript abundance was increased three-fold after 30 min, 1.5-fold after 60 min, and two-fold after 180 min compared to the control values. On the contrary, the 800 ppm CO_2_ treatment reduced *NtAQP1* expression compared to the control at all measurement times (Figure 2A).

Leaves fed with 400 ppm CO_2_ for 60 and 180 min showed a similar *NtPIP2;1* transcript accumulation, about 20% lower than that measured after 30 min from starting the treatment. Treatments with air containing 200 and 0 ppm CO_2_ concentrations significantly increased the expression of *NtPIP2;1*, whereas a significant decrease in transcript levels compared to the control was observed in the 800 ppm CO_2_ treatment (Figure 2B).

## 3. Discussion

We have analysed the expression responses of two aquaporin genes in tobacco leaves treated with air containing different CO_2_ concentrations. Treatments were applied to the leaf patches enclosed by the gas exchange leaf chamber. The values of C_i_ were estimated using the model of von Caemmerer and Farquhar [33], which requires the input of P_n_. It has been shown that lateral CO_2_ movement in homobaric leaves (such as those of tobacco) can take place if a CO_2_ gradient is present and that this movement can cause P_n_ values which are not correctly measured by gas exchange [34,35,36]. Indeed, some of our treatments induced a sharp CO_2_ gradient across the boundary between the projection of the leaf chamber and the rest of the leaf, thus potentially inducing alterations of P_n_ which would have not be measured by gas exchange and thus could have caused errors in the assessment of C_i_. We are, however, confident that our C_i_ measurements reflected real intercellular CO_2_ concentrations as i) we used a relatively large leaf chamber (lateral CO_2_ movement extends in the order of a few millimeters [37]) and ii) the increases in P_n_ induced by lateral CO_2_ flow and not revealed by gas exchange measurement were not higher than 20% in leaf chambers *ca.* six times smaller than we used [34], and this would not radically change the C_i_ differences we measured between treatments.

**Figure 3:**
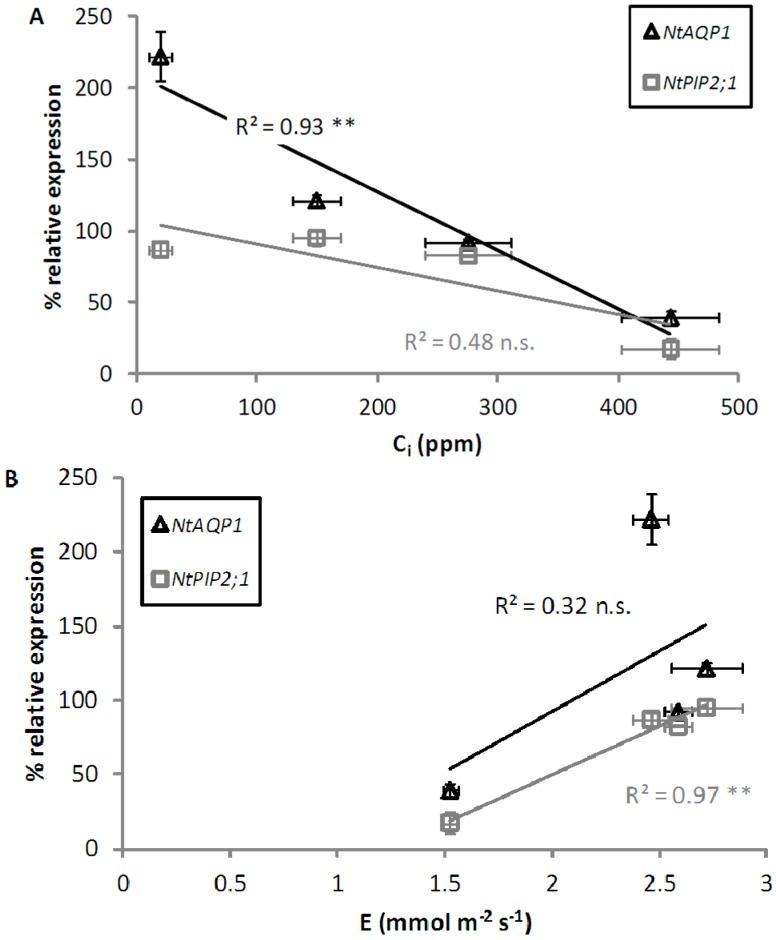
*NtAQP1* and *NtPIP2;1* transcript levels were plotted respectively *vs.* (**A**) leaf substomatal CO_2_ concentration (C_i_) and (**B**) transpiration rate (E). *NtAQP1*: black empty triangles; *NtPIP2;1*: grey empty squares (means ± SE, *n* = 3). Asterisks mark significance of regression (** *p* < 0.01, n.s., not significant).

Our results show that the expression of the aquaporin genes was inversely correlated to CO_2_ concentrations (Figure 3A). This relationship was relatively strict and significant for *NtAQP1*, and much less evident for *NtPIP2;1*. It is notable, even if physiologically not relevant, that at 0 ppm CO_2_, expression markedly increased compared to the control for the *PIP1* gene, while it was only weakly affected in the case of *NtPIP2;1*. A possible mechanistic explanation of these results is that aquaporin (and in particular *NtAQP1*) expression is directly controlled by the CO_2_ concentration in the mesophyll cells, which is in equilibrium with substomatal air [CO_2_]. A regulative role of CO_2_ on plant gene expression has been reported for genes involved in a range of processes such as primary metabolism [38], ripening of fruits [39,40], and development of floral organs [41]. At present, there is only sporadic information about modifications of aquaporin gene expression in response to changing CO_2_ air concentration. A microarray analysis study following six years of exposure of poplar to 550 ppm [CO_2_] in a FACE (free-air CO_2_ enrichment) experiment [26] reported downregulation of aquaporin genes belonging to both the *PIP1* and *PIP2* subfamilies. The expression decrease reported by these authors was less pronounced than in our experiment, possibly due to acclimation effects, and to the fact that air [CO_2_] was about 550 ppm in the FACE experiment, while we fed leaves 800 ppm CO_2_.

Exposure of leaves to different air [CO_2_], however, also affects stomatal conductance, leaf evaporation, and may thus potentially induce localized water stress. Some of these parameters control aquaporin expression and thus the effect of changing air [CO_2_] could be indirect and mediated by these factors. Aquaporin expression is affected by hyperosmotic stresses such as water, salt, and cold stress. Our plants were well watered and soil water availability was strictly controlled, in order to keep leaf water potentials always high. No treatment induced stomatal opening above values measured in control (400 ppm) leaves, and thus the potential induction of local areas of lower water potential within the leaf chamber should be ruled out. Some reports suggest that leaf evaporation may control leaf or shoot aquaporin expression [42,43], possibly through accumulation of ABA in the evaporating tissues [44]. In our experiment, E was positively correlated with aquaporin expression, especially in the PIP2 gene, suggesting that expression could be positively controlled by E, besides the negative control exerted by CO_2_ (Figure 3B). Interestingly, the maximum transcript level for *NtAQP1* was indeed achieved after 30 min at zero [CO_2_], when, due to the initial adaptation stage, E did not significantly differ among treatments.

Taking into account that NtAQP1, and not NtPIP2;1, shows CO_2_ transport facilitation properties [24], these observations fit in a model where upregulation of a CO_2_-transporting aquaporin is activated at low C_i_ and helps to maintain photosynthetic levels, while downregulation takes place in a situation where high C_i_ saturates photosynthesis and causes stomatal closure [25,45]. This pattern of regulation could have important functional implications in the facilitation of CO_2_ transport to the mesophyll cells. Transgenic over- or under-expression of aquaporins of the PIP1 and PIP2 subfamilies indeed affects g_m_ in barley, tobacco and *Arabidopsis* leaves [14,15,46]. Changes of mesophyll CO_2_ conductance (g_m_) have been analyzed by Flexas *et al.* [28] in the 200–1000 ppm C_i_ range on tobacco leaves with an experimental setup similar to ours. Their results have a striking similarity to the changes in aquaporin gene expression we observed, and modifications of *NtAQP1* expression in this experiment followed the same trend as g_m_ in the cited paper.

In conclusion, expression of *NtAQP1* negatively responds to air [CO_2_] in the whole range of 0–800 ppm. On the contrary, gene expression of *NtPIP2;1*, an aquaporin not facilitating CO_2_ transport, is little affected by air [CO_2_], and changes in parallel with transpiration. Our results suggest that expression of *NtAQP1* may be an essential determinant of plant adaptation to changing air [CO_2_]. Aquaporins act as molecular compensatory mechanisms to environmental constraints [47,48,49]. To our knowledge, this is the first example of a compensatory role at the transcript level related to changing CO_2_ availability. At the post-transcriptional level, it is known that aquaporins are gated off by low pH [50]; exposing cells to high CO_2_ is also expected to lower the cytoplasmic pH, and this helps to deactivate aquaporins at a high level of air [CO_2_].

## 4. Materials and Methods

### 4.1. Plant Materials, CO_2_ Treatment and Gas Exchange Measurements

The experiment was carried out on leaves of *Nicotiana tabacum* L. cv. Samsun. Seeds were planted in trays on soil and after four weeks the plants were transplanted and kept in a shaded greenhouse in 3 L containers filled with a substrate composed of a sandy-loam soil/expanded clay/peat mixture (3:1:2). Photosynthetic photon flux density (PPFD) in the greenhouse averaged 120 μmol·m^–2^·s^–1^ at the beginning of the experiment and ambient CO_2_ concentration was 390 ppm.

Twenty-five (±2.1)-day-old tobacco leaves from 15 plants were used for both gas exchange measurements and expression analysis. Leaf portions (6.25 cm^2^) enclosed in a sealed chamber were continuously fed with air (200 mL·min^–1^) containing different CO_2_ concentrations such that CO_2_ concentration within the leaf chamber was respectively 0, 200, 400 and 800 ppm (four treatments in total), using an LCpro+ portable gas exchange system (ADC Bioscientific, Great Amwell, UK). Each treatment was subsequently applied for 30, 60 and 180 min on three consecutive leaves on the same plant in a single day from 10 a.m. to 2:30 p.m. ± 0.06 h and each treatment was carried out on three plants (*n* = 3). Measurements were completed within 12 successive days following a randomized distribution of the four treatments and of the three biological replicates (plants).

Gas exchange measurements were performed using the same LCpro+ portable gas exchange system, based on an open-flow gas circuit system equipped with microclimate control. H_2_O and CO_2_ concentrations at the inlet and outlet of the cuvette were measured using a differential infrared gas analyzer. The leaf chamber area was 6.25 cm^2^ and the PPFD above the leaf portion enclosed within the chamber was 350 μmol·m^–2^·s^–1^ PPFD. Leaf temperature was 26.2 ± 0.32 °C throughout the measurement time. Data were recorded at 2 min intervals throughout the treatment time. Sub-stomatal CO_2_ concentration (C_i_) was calculated according to Farquhar *et al.* [33,45]. Data analysis and calculations were carried out using Microsoft Excel (Microsoft Corporation, Redmond, WT, USA).

### 4.2. RNA Extraction, cDNA Synthesis and Real Time PCR

Total RNA was extracted from three independent treated leaves collected from three plants from each treatment and (thus corresponding to three biological replicates for each treatment) according to the protocol described by Prescot *et al.* [51]. Further leaf samples (*n* = 3) were collected from three plants used as control for preliminarily monitoring the gene expression level at ambient [CO_2_], during a time span of 4.5 h (from 10 a.m. to 2:30 p.m.).

RNA yield and purity were determined spectrophometrically at A260 and A280, and its integrity checked by electrophoresis on an agarose gel. Contaminant genomic DNA was removed from the samples by digestion with RNase-free DNase I (Fermentas). cDNA was synthesized using SuperScript II Reverse Transcriptase (Invitrogen) according to supplier’s instruction, and the resulting cDNA was diluted and used as a template in PCR reactions.

Primer 3 program [52] was used to design specific primers: the forward primers were designed on the open reading frame (ORF) regions while the reverse primers on 3′-untranslated (UTR) regions. Primers were characterized by a length of 20–24 nucleotides, a predicted melting temperature (T_m_) of 60–63 °C, and amplicon lengths of 100–130 base pairs (bp). The primer sequences used for gene expression analysis are listed in the Table 1.

Transcript abundance for NtAQP1 (GenBank AJ001416) and NtPIP2;1 (GenBank AF440272) genes in the leaves exposed to various CO_2_ treatments was quantify by real-time PCR with iQTM SYBR Green Master Mix (Bio-Rad, Hercules, CA, USA) on an ICycler Q apparatus (Bio-Rad, Foster City, CA, USA). Reactions were done in 20 μL final volumes containing 0.5 μM of each primer, 2 μL of cDNA appropriate dilution and 10 μL of 2× iQ™ SYBR Green Master Mix Reagent (Bio-Rad; containing 100 nM KCl, 40 mM Tris-HCl, pH 8.4, 0.4 mM dNTPs, 50 U/μL iTaq DNA polymerase, 6 mM MgCl_2_, 20 nM SYBR Green I, 20 nM fluorescein). PCR cycling program consisted of one cycle of 2 min at 95 °C, followed by 45 cycles of 95 °C for 15 s and 60 °C for 45 s, with a final melt gradient starting from 50 °C and heating to 95 °C at a rate of 0.5 °C·s^–1^. The efficiency of the primer set was evaluated by performing standard curve with five dilutions of cDNA and similar values were obtained.

The resulting data were analyzed using ICycler software (Bio-Rad, Foster City, CA, USA), and the values were normalized to the transcript levels of elongation factor 1α (*EF1*α) gene. In order to evaluate the stability of *EF1α* transcript abundance and its suitability as a housekeeping control, gene expression values were also normalized using actin as the reference gene. No significant changes were observed when data were normalized with any of the two different reference genes (data no shown). RT-PCR was carried out using three biological replicates for treatment and time; and three technical replicates were performed for each of the three biological sample.

The data were organized according to the “comparative threshold cycle” method [53] and the relative expression level of each gene in different conditions was referred to that of a calibrator gene set to 100 and represented by the expression value of leaves subjected to 400 ppm air [CO_2_] at time 0 (control samples).

### 4.3. Statistical Analysis

Data were analyzed with the Sigma Stat 2.0 (SPSS, Chicago, IL, USA) statistics 16 package. One-way ANOVA was used to test differences between experimental groups. We used Tukey’s test to make *post-hoc* pair-wise comparisons between means. Samples from leaves subjected to 400 ppm air [CO_2_] were considered as ambient control samples.

## 5. Conclusions

Gene expression for *NtAQP1* positively responds to CO_2_ scarcity in the air in the whole range of 0–800 ppm. On the contrary, gene expression for NtPIP2;1, an aquaporin not facilitating CO_2_ transport, is related to water balance in the leaf, and changes in parallel with stomatal conductance.

Our results suggest that expression of *NtAQP1* and *NtPIP2;1* is an essential determinant of mesophyll conductance in tobacco under changing C_i_. Further research is needed to verify whether g_m_ and aquaporin expression are coupled also under changes in other environmental and physiological parameters. Aquaporins are known to act as a molecular compensatory mechanism of morphological and/or functional constraints [47,48,49]. However, to our knowledge, this is the first example of a compensatory enhancement of aquaporin expression related to changing CO_2_ availability.

Our observations could fit in a model where upregulation of CO_2_-transporting aquaporins can be activated at low internal [CO_2_] (C_i_), thus helping to maintain photosynthetic levels, while down-regulation takes place in a situation where high C_i_ saturates photosynthesis and causes stomatal closure. As the expression of aquaporin genes in leaves addresses plant regulation upon abiotic stress [44,54], the aquaporin-specific control on water *versus* carbon pathways in leaves [30] will possibly drive future research in this topic [55].

## Figures and Tables

**Table 1:** List of primers used for quantitative Real Time PCR.

Gene Name	Forward Primer (5′–3′)	Reverse Primer (5′–3′)
*NtAQP1*	CTGGATCTTTTGGGTTGGAC	CAGAAAGATTAAGGCTTCTTGAGG
*NtPIP2;1*	CATTTGTGGGAGCATTGGTA	CTGGTAGTGGTTGCAAAAGTTG
*NtEF-1α*	CTCTCTGCGTACCCACCATT	TAGCACCAGTTGGGTCCTTC
*Actin*	CGTCCTTAGTGGTGGAACA	GCCACCACCTTGATCTTC

## Abbreviations

C_i_: leaf internal (substomatal) CO_2_ concentration
G_s_: stomatal conductance
P_n_: leaf net photosynthesis
E: transpiration rate
ANOVA: analysis of variance
